# Alteration of histone H3K4 methylation in glomerular podocytes associated with proteinuria in patients with membranous nephropathy

**DOI:** 10.1186/s12882-016-0390-8

**Published:** 2016-11-17

**Authors:** Takayuki Fujino, Naoyuki Hasebe

**Affiliations:** Department of Internal Medicine, Cardiovascular Respiratory and Neurology and Nephrology Division, Asahikawa Medical University, Midorigaoka Higashi 2-1-1-1, Asahikawa, 078-8510 Japan

## Abstract

**Background:**

Histone H3K4 trimethylation (H3K4 me3) is found in active euchromatic regions and plays an important role in podocyte function in which actin filaments are abundant in the foot processes. The pathogenesis of membranous nephropathy (MN), the most prevalent cause of primary nephrotic syndrome in the middle-aged and elderly, is podocyte dysfunction.

**Methods:**

We investigated the role of H3K4 me3 in podocyte dysfunction in nephrotic syndrome using cultured podocytes and a mouse proteinuria model induced by LPS. We examined renal biopsy specimens from six patients with nephrotic syndrome caused by Phospholipase-A2-Receptor-positive primary MN.

**Results:**

H3K4 me3 exhibited a pattern of nuclear expression in podocytes of the kidneys from patients with MN. The overlapping expression of H3K4 me3 and cathepsin L (a potent endoprotease causing the breakdown of actin-associated protein within lysosomal compartments in kidney podocytes) were higher in patients with MN compared with the controls. Histone H3K4 me3 in kidney podocytes was negatively correlated with synaptopodin, an actin-associated protein in podocytes, and the expression was positively correlated with the proteinuria levels in patients with MN. Histone H3K4 me3 levels were elevated in podocytes of LPS-treated mice, combined with an increase in podocyte swelling, an elevation of serum creatinine and urine albumin, increased cathepsin L, and decreased synaptopodin expression. Histone H3K4 me3 levels at the cathepsin L promoter were elevated in LPS-exposed mouse kidneys. The administration of shRNA against MLL3 (an H3K4 methyltransferase) to LPS-treated mice and cultured podocytes co-cultured with LPS-stimulated macrophages ameliorated podocyte swelling, an elevation in the serum creatinine and urine albumin levels and an increased expression of histone H3K4 me3 and cathepsin L, and a decreased expression of synaptopodin and increase in histone H3K4 me3 levels at the cathepsin L promoter.

**Conclusions:**

Histone H3K4 me3 upregulation may be involved in podocyte dysfunction and the pathophysiology of MN. Targeting this epigenetic signature of histone H3K4 me3 followed by modulating the actin dynamics may be an effective strategy to ameliorate the consequences of MN.

## Background

The median age of onset of membranous nephropathy (MN) is in the early 50s, and is the most important cause of the nephrotic syndrome in the elderly (aged > 65 years). MN pathogenesis involves podocyte injury caused by viruses, drugs, or autoimmunity deficiency. Podocytes are the largest cells in the glomerulus and play a central role in the glomerular filtration barrier of the kidney [1, 2]. Podocytes contain a well-developed Golgi complex, with lysosomes containing proteasomal enzymes (e.g., cathepsin L) [3]. Large numbers of microtubules, microfilaments, and intermediate filaments are present in the cytoplasm, and actin filaments are particularly abundant in the foot processes where they connect the slit membrane with the glomerular basement membrane. Several studies have described molecular changes in the podocytes of patients with MN [2, 4, 5].

Histone modifications are key changes in the chromatin architecture that correlate with the changes in gene expression. The closed chromatin template is inaccessible to regulatory cofactors and thus is transcriptionally suppressed. In contrast, the open conformation enables the entry and recruitment of key transcriptional enzymes, such as RNA pol II. Trimethylation of histone H3 on lysine 4 (H3K4 me3) is found in active euchromatin but not in silent heterochromatin [6, 7].

To investigate the role of H3K4 me3 in the pathophysiology of nephrotic syndrome, we examined renal biopsy specimens from six patients with nephrotic syndrome that was caused by primary MN. We studied H3K4 me3 in a mouse proteinuria model induced by LPS and in cultured podocytes.

## Methods

### Subjects and samples

Archival renal biopsy tissue, serum, and urine samples were available from six patients with primary MN. The tissues were classified by stage according to the Ehrenreich-Churg classification system using transmission electron microscopy, and the immunohistochemical differences between the three stages were determined. Cryosections of kidney biopsy specimens were obtained from patients with MN and stained with anti-human IgG, IgA, IgM, C3, C4, and C1q antibodies (Dako, USA). Sections (4 μm) embedded in paraffin were stained with hematoxylin and eosin, periodic acid-Schiff stain, periodic acid-methenamine-silver stain, Masson trichrome stain, and immunohistochemically analyzed as described below. All subjects provided informed written consent. This study was approved by the Asahikawa Medical University Institute Ethics Committee. Patients with minimal change (*N* = 2), focal segmental glomerular sclerosis (FSGS, *N* = 2), and AL amyloidosis (*N* = 3) were examined as the human controls of patients with other causes of nephrotic syndrome. Three controls were subjected to a renal biopsy with finding of microhematuria without proteinuria and no pathological findings of glomerulonephropathy (control A (ConA)). Another age-matched control was used as a frozen and paraffin tissue section of a healthy human adult normal kidney (control B (ConB), BioChain Institute, Inc., CA, USA).

### Histological analysis

The kidneys were fixed in a solution of buffered paraformaldehyde, embedded in paraffin, and sectioned (4 μm) in an LPS-induced proteinuria model. For immunohistochemistry, paraffin-embedded kidney sections from human subjects and mice were incubated with antibodies against nephrin (NOVUS BIOLOGICALS, Littleton, CO, USA), histone H3K4 me3 (EPIGENETEK, Farmingdale, NY, USA), cathepsin L (R&D Systems, Inc., Minneapolis, MN, USA), Wilm’s tumor-1 protein (WT-1, DAKO, USA), synaptopodin (PROGEN Biotechnik, Heidelberg, German), and anti-GFP (anti-GFP-tag, rabbit polyclonal antibody; AnaSpec, Inc., Fremont, CA, USA), NEPH-1 (KIRREL1, BIOSS ANTIBODIES, Woburn, MA, USA), phospholipase A2 receptor 1 (Anti-PLA2R1, ATLAS ANTIBODIES, Stockholm, Sweden), followed by an incubation with a biotinylated secondary antibody and streptavidin-HRP. Tissue sections were visualized using TACS Blue Label (Trevigen Inc., Gaithersburg, MD, USA) to generate a dark blue color. For immunofluorescence, Alexa Fluor 594 goat anti-mouse IgG (H + L) (ab150116; Abcam, Cambridge, UK), Alexa Fluor 488 donkey anti-rabbit IgG (H + L) (A21206; Life Technologies, Carlsbad, CA, USA), Alexa Fluor 594 goat anti-rat IgG (H + L) (ab150160; Abcam), Alexa Fluor 594 donkey anti-goat IgG (H + L) (ab150132; Abcam), Alexa Fluor 594 donkey anti-rabbit IgG (H + L) (ab150076, Abcam), Alexa Fluor 488 goat anti-mouse IgG (H + L) (ab150113, Abcam) for the secondary antibody and a fluorescence microscope for visualization (BZ-X700, KEYENCE, Osaka, Japan) were used.

Glomerular images (4–10) from mouse and human kidneys were captured with a 40× objective for each case. All of the regions of interest (ROIs) in each glomeruli were measured automatically, after which the results were normalized for each glomerular area, and the percent area was determined using the image analyzer (KEYENCE, Osaka, Japan). All final measurements used the mean values of 4–10 glomerular images captured from each case. The mean ± standard error of three measurements of the merged yellowish areas of H3K4 me3 and cathepsin L staining in the same glomeruli of the control was 0.70% ± 0.16%.

### Transmission electron microscopy

Renal tissue was incubated with 4% paraformaldehyde for 2 h and embedded in LR white resin (London Resin Company Limited, London, UK). Ultrathin sections were prepared using a diamond knife (DIATOME, Switzerland) and microtome (Sakura Co. Ltd, Tokyo, Japan). The sections were mounted on a grid mesh coated with polyvinyl formal (Okenshoji Co Ltd, Tokyo, Japan) and dual-stained with uranyl acetate in 10% ethanol (w/v) and lead citrate in 0.1% (w/v) 0.1 M NaOH. Analyses were performed using a transmission electron microscope (Hitachi, Tokyo, Japan). The podocyte cell area was measured using the image analyzer (Hitachi, Tokyo, Japan).

### Western blotting

Kidney tissues from mice with an LPS-induced model of proteinuria were weighed, divided into pieces that included the cortex using a clean razor blade, and stored in Allprotect Tissue Reagent (QIAGEN, Venlo, Limburg, Netherlands). The tissue was then disrupted and homogenized using a Dounce homogenizer (TissueLyser, QIAGEN) and transferred to microcentrifuge tubes. RNA, DNA, and protein were extracted using the AllPrepDNA/RNA/Protein Mini kit (QIAGEN). Equal amounts of protein as determined using the bicinchoninic acid method (Thermo Fisher Scientific K.K, MA, USA) were diluted in SDS sample buffer [62.5 mM Tris/HCl (pH 6.8), 1.25% (w/v) SDS, 25% (w/v) glycerol] and boiled for 2–3 min. Samples (10 μg/10 μL) were then loaded onto polyacrylamide gels (ePAGEL; ATTO, Tokyo, Japan). ECL DualVue markers (GE Healthcare Bio-Sciences AB, Uppsala, Sweden) were used as molecular weight standards. After electrophoresis (AE-6351 PageRun, ATTO), the gels were transferred to a Hybond-P PVDF (Immobilon-P, 0.04 mm; EMD Millipore Corporation, Billerica, MA, USA) using wet transfer equipment (Mini-PROTEAN Tetra; Bio-Rad, Tokyo, Japan). Nonspecific binding was blocked by incubating the membrane in Amersham ECL Prime Blocking Agent (RPN418) or 5% nonfat dried milk and 0.1% (v/v) Tween 20 in TBS (TBS-T) for 1 h at room temperature in a covered container. The membrane was rinsed by changing the wash buffer twice. The blocked membrane was incubated overnight at 4 °C in blocking buffer containing the following primary antibodies: histone H3K4 me3 (Epigentek, Farmingdale, NY, USA), cathepsin L (R & D Systems, Inc., Minneapolis, MN, USA), and synaptopodin (p-19; Santa Cruz Biotechnologies, Santa Cruz, CA, USA). The membrane was washed for 15 min three times in TBS-T, followed by incubation for 60 min at room temperature with a horseradish peroxidase (HRP)-conjugated secondary antibody (GE Healthcare Bio-Sciences AB) diluted 1:10,000 in wash buffer. The membrane was washed for 15 min three times with TBS-T and visualized using the ECL Prime Western Blotting Detection System (GE Healthcare Bio-Sciences AB). Images were captured using a CCD camera (LAS-3000, GE Healthcare).

### Determination of H3K4 me3 levels from acid extraction material

H3K4 me3 levels were determined using a material isolated by acid extraction (The EpiQuik™ Total Histone Extraction Kit, EPIGENETEK, Farmingdale, NY, USA), and global tri-methylation of histone H3-K4 was measured using the EpiQuik Global Tri-Methyl Histone H3-K4 Quantification Kit determined by a sandwich enzyme-linked immunosorbent assay (ELISA). The EpiQuik Total Histone H3 Quantification Kit (EPIGENETEK, sandwich ELISA) was used for measuring the modified histone H3 content of the samples for standardization.

### LPS-induced model of proteinuria

Male C57BL/6 mice weighing 15–20 g were housed in standard cages in climate- and light-controlled rooms (12 h light–dark cycle) at 22 °C with free access to food and water. All animal studies were approved by the Asahikawa Medical University Animal Institute Committee. We induced proteinuria in male mice via an LPS injection as previously described [8]. Mice were injected with ultrapure LPS (100 μg intraperitoneally [i.p.]) (LPS-EK ULT Escherichia coli K12, Invivogen, San Diego, CA, USA). After 24 h, the LPS administration (50 μg) was repeated, and shRNA was injected as described below. The mice were sacrificed 24 h after shRNA injection. Urine and plasma samples were assessed for albumin (QuantiChrom BCG Albumin Assay Kit, BioAssay Systems, Hayward, CA, USA) and creatinine levels (QuantiChrom Creatinine Assay Kit, BioAssay Systems, Hayward, CA, USA) according to the manufacturer’s protocols. Albuminuria in mice was determined as arbitrary units of albumin per milligram of creatinine.

### In vivo gene delivery of shRNA targeting MLL3

In vivo hydrodynamic gene delivery was achieved as previously described (1), with some modifications in an LPS-induced model of proteinuria. We then inoculated the mice (*N* = 10 per group) with 100 μg LPS i.p., and 24 h after administration, we administrated another 50 μg LPS i.p. and injected anti-nephrin antibody-flag-shRNA through the penile vein. The MaxCarrierT3 Conjugation Kit (Bioo Scientific Corporation, Austin, TX, USA) was used to conjugate the T3 carrier to an antibody against nephrin specific to an extracellular protein domain on podocytes (R&D Systems, Inc., Minneapolis, MN, USA). A mixture of 20 μg shRNA against MLL3, a control, and a plasmid-encoding GFP was added to the T3 conjugate along with in vivo-jet PEI cationic polymer transfection reagent (1 μL) (Polyplus-transfection Inc., New York, NY, USA) in a 5% glucose solution to yield a total volume of 200 μL. All shRNA molecules used in this study were synthesized by Santa Cruz Biotechnology. Three target-specific lentiviral vector plasmids, each encoding 19 nt–25 nt (plus a hairpin) shRNAs, were designed to knockdown the gene expression. These plasmids are as follows: MLL3 shRNA plasmid (m: sc-29436-SH), control plasmid (sc-108060), and copGFP Control Plasmid (sc-108083). The copGFP control plasmid is a lentiviral vector plasmid encoding the copGFP fluorescent protein in mammalian cells. The control plasmid encodes a nonfunctional protein. Following the in vivo transfection, the efficacy of the gene transfer was assessed by an immunohistochemical analysis of GFP and the podocyte markers nephrin and NEPH-1.

### Chromatin immunoprecipitation assay

Molecular events in the kidneys of LPS-treated mice were monitored using a chromatin immunoprecipitation (ChIP) assay to immunopurify soluble chromatinized H3K4 me3 sequences using specific antibodies and the EpiQuik ChIP kit (EPIGENTEK, Brooklyn, NY, USA). The antibodies used included normal mouse IgG as the negative control, anti-RNA polymerase II as the positive control, and anti-H3K4 me3 (EPIGENTEK, Brooklyn, NY, USA). The DNA concentration was quantified using a NanoDrop UV spectrometer (NanoDrop Technologies, Wilmington, DE, USA). A SYBR green assay (LightCycler 480 SYBR Green I Master; Roche, Mannheim, Germany) was performed using the 7300HT Fast Real-Time PCR System (Applied Biosystems, Carlsbad, CA, USA) using primers for mouse cathepsin L (EpiTect ChIP qPCR Assays, QIAGEN).

### Podocyte culture

Conditionally immortalized mouse podocytes (podocyte cell line E11) cloned from the outgrowth of glomeruli isolated from H-2 kb-tsA58 transgenic mice were purchased from Cell Lines Service (CLS cell line service, Eppelheim, Germany). Podocytes were cultured according to the method of Endlich et al. [9]. Undifferentiated podocytes were cultured in RPMI1640 medium (GIBCO, USA) with 10% fetal bovine serum (FBS) (GIBCO) in an incubator at 33 °C in 5% CO_2_. Podocytes were cultured at 38 °C in RPMI1640 medium for a minimum of 14 days to allow for complete differentiation [10].

Lentiviral vector plasmids each encoding MLL3 shRNAs (sc-62624-SH; Santa Cruz Biotechnology) and a transfection reagent (sc-108061) were used for the inhibition of MLL3 expression in E11 cells. Lentiviral vector plasmids each encoding GFP shRNA (sc-45924-SH; Santa Cruz Biotechnology) were used to confirm the efficiency of the transfection. After transduction, the E11 line expressing the shRNA was used for the Western blot analysis and acid extraction of histones followed an ELISA assay for H3K4 trimethylation and ChIP assay.

### Isolation and co-culture of peritoneal macrophages with podocytes

Peritoneal macrophages (pMACs) were isolated from B57CL/6 mice. A volume of 2 mL of 3% thioglycollate (Sigma-Aldrich, USA) was injected into the abdominal cavity to activate pMACs and thus, enable the harvest of an optimal yield. After 3 days, the mice were killed with an overdose of sodium pentobarbital via a subcutaneous injection. Then, 10 mL of cold sterile RPMI 1640 was injected intraperitoneally, and peritoneal lavage fluids were collected using sterile syringes. For juxtacrine co-cultures, pMACs were seeded in a Falcon® cell culture insert for six-well plates (0.4 μm, high density pore, 1 × 10^5^ cells/well, Corning, NY, USA). After incubation at 37 °C for 4 h to allow the pMACs to adhere to the surface of the cell culture insert, the non-adherent cells were removed. These cell culture inserts were placed into six-well plates seeded with podocytes (1 × 10^6^ cells/well) after the trans induction of control shRNA or MLL3 shRNA. These juxtacrine co-cultured cells were incubated with serum-free RPMI1640 (control), or serum-free RPMI1640 with 2 μg/mL lipopolysaccharide (LPS). At 24 h, podocytes from control or LPS groups transfected with each control or MLL3 shRNA were subjected to an H3K4 me3 assay, Western blot, and ChIP assay.

### Statistical analysis

Statistical comparisons of data were made using a one-way repeated-measurements ANOVA followed by a Dunnett’s test for multiple comparisons. The Mann-Whitney U test was used to compare variables between the two groups. The results are expressed as the mean ± standard error (SE). Analyses were performed using add-in statistical software from Excel (Ekuseru Toukei 2010, Social Survey Research Information Co., Ltd., Tokyo Japan). A two-tailed probability value < 0.05 was considered to be statistically significant.

## Results

### Distribution of histone H3K4 me3 in human primary acquired glomerular diseases

**Fig. 1:**
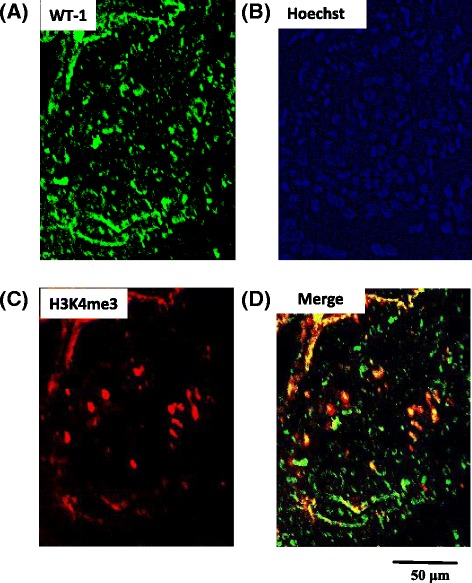
Localization of trimethylated histone H3K4 in human kidney glomeruli. Immunohistochemical staining indicates the co-localization of trimethyl-histone H3K4 (H3K4 me3) with the podocyte marker WT-1. The overlapping staining for WT-1 (*green*, **a**) and H3K4 me3 (*red*, **c**) results in a merged image (*yellowish*, **d**); nuclei are stained blue using Hoechst 33342 (**b**)

H3K4 me3 exhibited a pattern of nuclear expression in the podocytes of kidneys from patients with membranous nephropathy (MN; Fig. 1). The co-localization of H3K4 me3 (Fig. 1c) and Wilm’s tumor-1 (WT-1) (Fig. 1a), a podocyte marker, was demonstrated by double immunofluorescence (Fig. 1d) in the glomeruli of patients with MN. H3K4 me3 may also be localized in mesangial cells and endothelial cell nuclei in the glomeruli.

### Pathophysiological role of histone H3K4 me3 in patients with MN

**Fig. 2:**
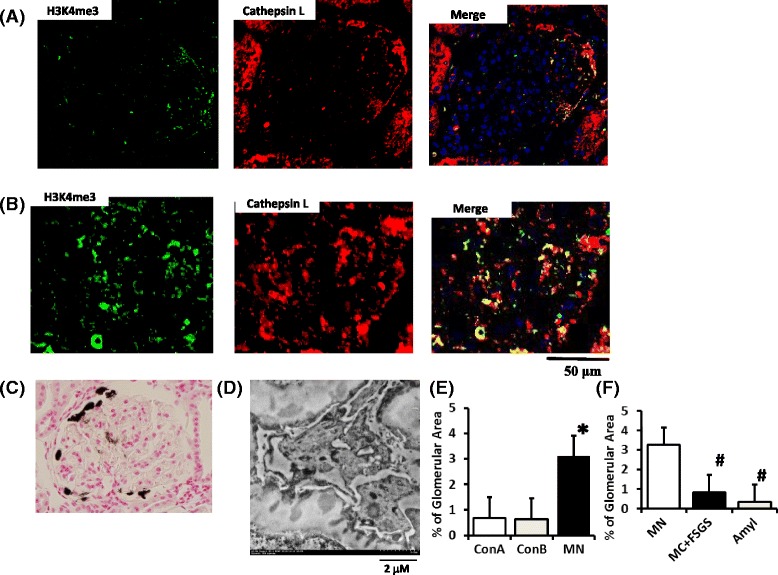
Expression of trimethylated histone H3K4 and cathepsin L in a patient with membranous nephropathy. Immunohistochemical staining indicates the overlapping staining for H3K4 me3 (*green, left side*) and cathepsin L (*red, center*) resulting in the merged image (*yellowish, right side*) in the control (**a**) and MN patient (**b**). Biopsies showing primary MN were diagnosed by positivity for PLA2R1 staining (**c**, Case 1 in Table 1) and subepithelial deposits according to electron microscopy findings (**d**, Case 1 in Table 1). The overlapping expression of H3K4 me3 and cathepsin L in patients with MN (*N* = 6) was significantly higher than in those of the ConA or ConB (**e**). ConA, control obtained in a series of consecutive patients (*N* = 3); ConB, age-matched healthy control, which were not in a series of consecutive patients (*N* = 3) (**e**). The overlapping expression of H3K4 me3 and cathepsin L in patients with MN was significantly higher than that of minimal change or FSGS or amyloidosis (**f**). H3K4 me3, histone H3K4 trimethylation; MN, membranous nephropathy; MC, minimal change; FSGS, focal segmental glomerular sclerosis; Amyl, AL amyloidosis. *P < 0.05 vs. ConA; #*P* < 0.05 vs. MN. Data are expressed as the mean ± SE

The expression of H3K4 me3 was evaluated immunohistochemically in patients with nephrotic syndrome who were diagnosed with primary MN (Table 1). None of the patients had received immunosuppressive therapy before the renal biopsy. Primary MN was diagnosed by immunostaining of the phospholipase-A2-receptor (PLA2R1) (Fig. 2c, Case 1 in Table 1) and sub-epithelial deposits as determined by electron microscopy (Fig. 2d, Case 1 in Table 1). All biopsies exhibiting membranous histology were graded by disease stage as estimated by the electron microscopy findings. Staining for H3K4 me3 was observed in the podocytes from the biopsies of all six patients with MN, with the intensity of staining varying between specimens.

**Table 1:** Characteristics of patients with primary membranous nephropathy

Case	Age	Sex	sCre	M-alb/cre (mg/g cre)	Stage	HT	Duration of disease prior to biopsy (months)	Hematuria	Previous Treatment
MN1	65	M	0.65	2931	II	+	24	+/−	ARB
									CCB
									Statin
MN2	78	M	0.95	1120	III	+	12	2+	ARB
									CCB
MN3	72	M	0.86	8188	I	-	8	3+	-
MN4	65	M	1.25	4306	II	-	4	2+	-
MN5	46	M	0.93	4136	I	+	2	3+	ARB
MN6	79	M	0.90	3168	I	+	6	2+	ARB
									CCB
									Statin
ConA1	33	M	0.60	12	-	-	-	2+	-
ConA2	27	F	0.60	7.6	-	-	-	1+	-
ConA3	46	M	0.98	6.2	-	-	-	2+	-

*sCre* serum creatinine (mg/dL), *stage* the stage of disease according to the Ehrenreich–Churg classification, *ARB* angiotensin II type 1 receptor blocker, *CCB* calcium channel blocker, *ConA1-3* three controls in a series of consecutive patients, which were subjected to a renal biopsy with finding of microhematuria without proteinuria and no pathological findings of glomerulonephropathy

The expression of H3K4 me3 and cathepsin L in patients with control (ConA, *N* = 3) and MN (*N* = 6) in a series of consecutive patients were examined (Table 1). Staining for H3K4 me3 (left side in Fig. 2a) was observed in the podocytes from the biopsies of the controls, in which H3K4 me3 was found to be co-localized with cathepsin L in the glomeruli (center and right side in Fig. 2a). The overlapping expression of H3K4 me3 and cathepsin L in patients with MN (Fig. 2b and e) was significantly higher than that of the controls (ConA, Fig. 2a and e). The expression of H3K4 me3 and cathepsin L in patients of other age-matched controls, which were not a series of consecutive patients (ConB, *N* = 3, Table 2) and MN (*N* = 6, Table 1) were examined. The overlapping expression of H3K4 me3 and cathepsin L in patients with MN was significantly higher than that of the ConB (Fig. 2e).

**Table 2:** Characteristics of patients with nephrotic syndrome and healthy controls

Case	Age	Sex	sCre	M-Alb/cre (mg/g cre)	HT	Duration of disease prior to biopsy (months)	Hematuria	Previous Treatment
FGS 1	45	M	0.61	6087	+	1	2+	ARB
								Statin
FGS 2	47	M	3.50	4338	+	12	1+	ARB
								CCB
								statin
MC 1	24	M	0.71	3376	-	1	2+	-
MC 2	27	M	0.65	2868	-	1	1+	-
Amy1	73	M	0.83	4680	-	4	1+	-
Amy 2	82	F	0.65	5381	+	1	2+	ARB
								Statin
Amy 3	72	F	0.9	3600	-	48	2+	ARB
ConB1	44	M	-	-	-	-	-	-
ConB2	66	M	-	-	-	-	-	-
ConB3	78	M	-	-	-	-	-	-

*sCre* serum creatinine (mg/dL), *ARB* angiotensin II type 1 receptor blocker, *CCB* calcium channel blocker, *MC* minimal change, *FGS* focal segmental glomerular sclerosis, *Amy* AL amyloidosis, *ConB1-3* three age-matched control, which were not obtained in a series of consecutive patients

The overlapping expression of H3K4 me3 and cathepsin L in patients with nephrotic syndrome diagnosed as minimal change (*N* = 2) and FSGS (*N* = 2) and amyloidosis (*N* = 3) were examined (Table 2). The overlapping expression of H3K4 me3 and cathepsin L in patients with MN was significantly higher than in those with MC, FSGS, and alyloidosis (Fig. 2f).

**Fig. 3:**
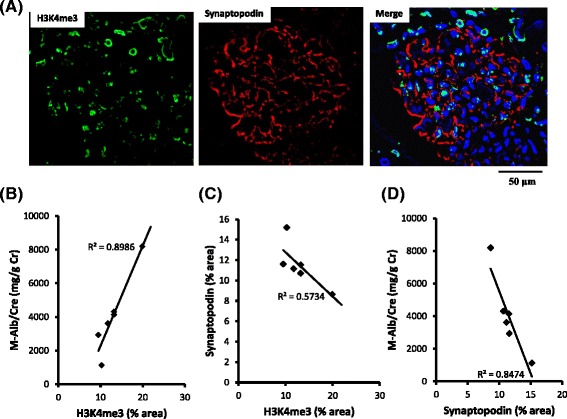
Association between histone H3K4 me3 and the expression of synaptopodin and proteinuria in patients with MN. Expression of H3K4 me3 (*left side* in **a**), synaptopodin (*center* in **a**), and the merged image (*right side* in **a**) are shown. The level of H3K4 me3 expression was positively correlated with albuminuria (**b**), and the level of H3K4 me3 expression was inversely correlated with the level of synaptopodin expression in the podocytes of patients with MN (**c**). The level of synaptopodin expression was inversely correlated with the level of albuminuria (**d**). MN, membranous nephropathy; H3K4 me3, histone H3K4 trimethylation

The expression of H3K4 me3 and synaptopodin were examined in the controls and patients with MN (Fig. 3a). As shown in Fig. 3b, a positive correlation was observed between the intensity of H3K4 me3 staining and the extent of proteinuria in patients with primary MN. The expression levels of synaptopodin in the podocytes were inversely correlated with the H3K4 me3 expression (Fig. 3c) as well as proteinuria, consistent with previous reports [11] (Fig. 3d). Thus, the overexpression of H3K4 me3 in podocytes was associated with the downregulation of synaptopodin, an actin-associated protein, and with increased proteinuria in patients with MN. The enhanced expression of PLA2R1 showed no significant correlation with the degree of proteinuria or H3K4 me3 expression (data not shown). The intensity of H3K4 me3 staining did not directly correlate with the stage of disease according to the Ehrenreich-Churg classification (data not shown).

### Elevated histone H3K4 me3 levels in the podocytes of kidneys from LPS-treated mice

**Fig. 4:**
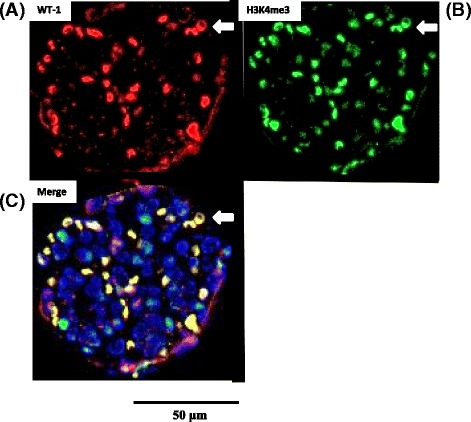
Localization of histone H3K4 me3 in mouse kidney glomeruli. Immunohistochemical staining indicates the co-localization of trimethyl-histone H3K4 (H3K4 me3) with the podocyte marker WT-1. The overlapping staining for WT-1 (*red*, **a**) and H3K4 me3 (*green*, **b**) results in the merged image (*yellowish*, **c**) as indicated by the *arrow*

**Fig. 5:**
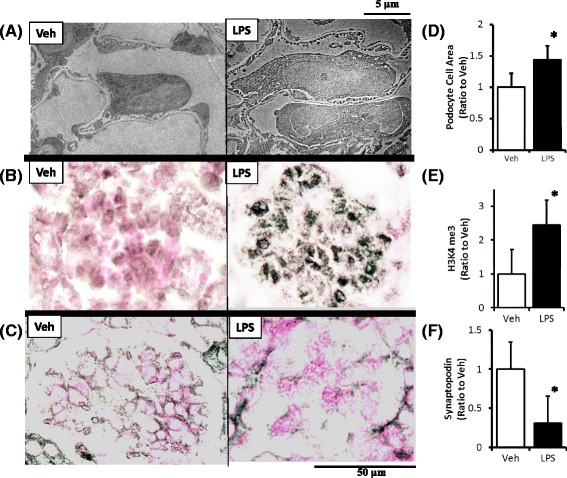
Podocyte swelling and the expression of H3K4 me3 and synaptopodin in the LPS model. Electron microscopy reveals podocyte swelling induced by LPS administration (*right side* in **a** and **d**) compared with the controls (vehicle alone, *left side* in **a** and **d**). The expression of histone H3K4 me3 was significantly higher following LPS administration (*right side* in **b** and **e**) than in the controls (vehicle alone, *left side* in **b** and **e**). Synaptopodin expression was significantly lower following LPS administration (*right side* in **c** and **f**) compared to that of the controls (vehicle alone, *left side* in **c** and **f**). *P < 0.05 vs. Veh. LPS, lipopolysaccharide treated mice; Veh, vehicle treated mice; H3K4 me3, histone H3K4 trimethylation; *N* = 10. Data are expressed as the mean ± SE

**Fig. 6:**
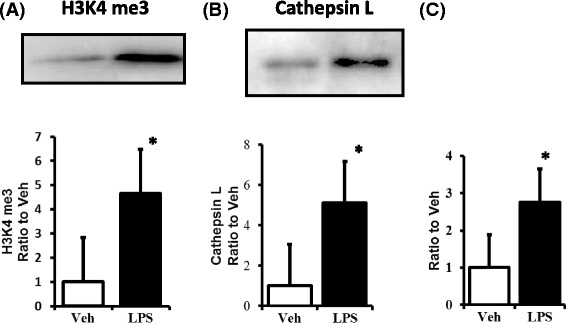
Expression of H3K4 me3 and cathepsin L and the determination of H3K4 me3 level on the promoter region of cathepsin L in the LPS model. Histone H3K4 me3 levels (**a**) and cathepsin L (**b**) levels were significantly higher in LPS-treated mice than in the controls (vehicle alone) as determined by a Western blot analysis. A chromatin-immunoprecipitation assay demonstrated that the amount of H3K4 me3 at the cathepsin L promoter was significantly higher in the LPS-treated mice than in the controls (vehicle alone) (**c**). *P < 0.05 vs. Veh. LPS, lipopolysaccharide treated mice; Veh, vehicle treated mice; H3K4 me3, histone H3K4 trimethylation; *N* = 10. Data are expressed as the mean ± SE

LPS-treated mice exhibited progressive proteinuria (vehicle, 0.50 AU/creatinine ± 0.69 AU/creatinine; LPS, 1.59 AU/creatinine ± 1.38 AU/creatinine). The immunohistochemical analysis revealed that H3K4 me3 was located primarily in the podocytes of LPS-treated mice (Fig. 4). LPS-treated mice exhibited podocyte swelling (Fig. 5a and d) similar to those observed with nephrotic syndrome. Immunohistochemical results confirmed that H3K4 me3 was upregulated in kidney podocytes 2 days following LPS administration (Fig. 5b and e). H3K4 me3 levels increased significantly over that of controls (vehicle alone) 2 days following LPS administration according to the Western blot analysis (Fig. 6a).

### Synaptopodin and cathepsin L levels in the kidneys of LPS-treated mice

Synaptopodin expression was analyzed immunohistochemically 2 days after LPS administration (Fig. 5c and f). Synaptopodin expression was significantly lower in LPS-treated mice than in the untreated controls (vehicle alone) (Fig. 5c and f). Cathepsin L expression was detected in podocytes using immunocytochemistry (data not shown). We next examined whether a similar upregulation of cathepsin L occurs in the LPS-treated murine model. As demonstrated by a Western blot analysis, cathepsin L levels were significantly higher in LPS-treated mice 2 days after administration than in the untreated controls (vehicle alone) (Fig. 6b).

### H3K4 me3 levels at the cathepsin L promoter in the kidneys of LPS-treated mice

To directly link H3K4 me3 to the cathepsin L locus, we conducted chromatin immunoprecipitation experiments to examine the changes in histone H3K4 me3 methylation patterns at the transcription initiation site of cathepsin L. The levels of H3K4 me3 at the cathepsin L promoter were significantly higher 2 days after LPS administration than in the untreated controls (vehicle alone) (Fig. 6c). These results suggest that the LPS-induced increase in cathepsin L expression is caused, at least in part, by epigenetic histone H3K4 me3 modifications at the promoter.

### Administration of MLL3 shRNA restored the LPS-induced upregulation of H3K4 me3 and cathepsin L and restored the downregulation of synaptopodin and podocyte swelling

**Fig. 7:**
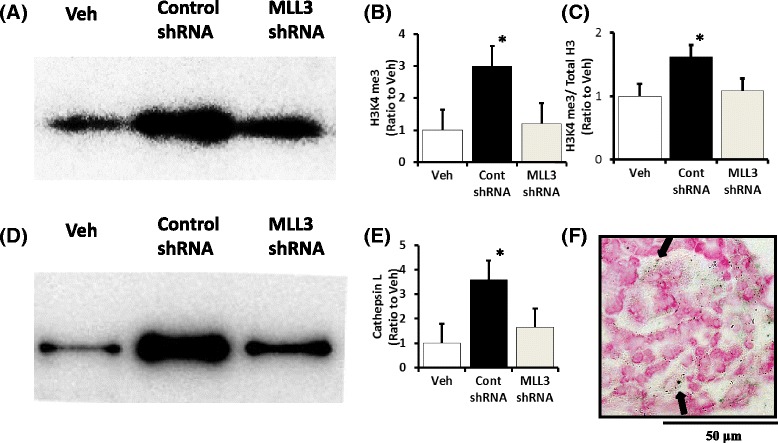
Effect of MLL3 shRNA on the expression of histone H3K4 me3 and cathepsin L in the LPS model. Expression of H3K4 me3 was determined by Western blot (**a** and **b**) and an ELISA using material isolated by acid extraction (**c**). The upregulated expression of H3K4 me3 (**a**–**c**) and cathepsin L (**d** and **e**) was significantly lower in MLL3 shRNA compared with control shRNA in the LPS-treated mice. GFP protein detected by immunohistochemistry using an anti-GFP antibody was clearly detected on the podocytes of kidneys transfected with GFP shRNA (**f**). *P < 0.05 vs. Veh. LPS, lipopolysaccharide treated mice; Veh, vehicle treated mice; H3K4 me3, histone H3K4 trimethylation; total H3, total histone H3; *N* = 10. Data are expressed as the mean ± SE

To determine whether H3K4 me3 was necessary for the altered protein expression observed in podocytes, we conditionally downregulated MLL3, an integral component of H3K4 methyltransferase, in the glomerular podocytes of mice. LPS-treated mice were transfected with 20 μg of shRNA targeting MLL3, a control, and GFP-containing plasmid. The efficacy of the shRNA transfection was determined on the basis of the transfection of GFP (Fig. 7f). GFP was detected by immunohistochemistry using an anti-GFP antibody that was expressed in the kidney podocytes from mice transfected with GFP shRNA (Fig. 7f), with an expression pattern similar to that of nephrin and NEPH-1 (data not shown) in the kidneys of LPS-treated mice.

The upregulation of H3K4 me3 was significantly inhibited by MLL3 shRNA compared with control shRNA as determined by Western blotting (Fig. 7a and b) and an ELISA using samples derived by acid extraction (Fig. 7c). The upregulation of cathepsin L was significantly inhibited by MLL3 shRNA compared with control shRNA determined by a Western blot (Fig. 7d and e).

**Fig. 8:**
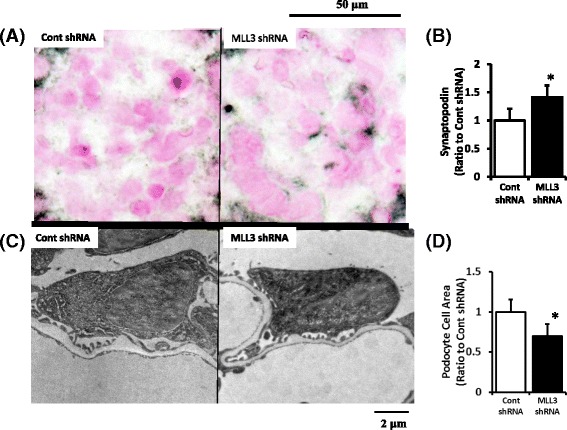
Effect of MLL3 shRNA on synaptopodin expression and podocyte swelling in the LPS model. LPS-induced decreased synaptopodin expression was significantly restored by MLL3 shRNA compared with control shRNA as detected by immunohistochemistry (**a** and **b**). Electron microscopy showed the suppressive effect of MLL3 shRNA on LPS-induced podocyte swelling (**c** and **d**). *P < 0.05 vs. control shRNA. LPS, lipopolysaccharide treated mice; Cont, control; *N* = 10. Data are expressed as the mean ± SE

The LPS-induced attenuation of synaptopodin levels was significantly restored by MLL3 shRNA compared with the control shRNA as detected by immunohistochemistry (Fig. 8a and b). Electron microscopy also showed this suppressive effect of MLL3 shRNA with respect to the podocyte swelling induced by LPS (Fig. 8c and d).

These results further support a key role of MLL3 in modulating H3K4 me3 to increase cathepsin L expression, decrease synaptopodin expression, increase podocyte swelling, and suggests a role of MLL3 shRNA in reversing the effects of LPS administration.

### Administration of MLL3 shRNA restored renal function and attenuated proteinuria

The urine albumin level, a marker of glomerular damage, was significantly lower in mice administered MLL3 shRNA than in those given the control shRNA (control shRNA, 2.05 AU/creatinine ± 0.60 AU/creatinine; MLL3 shRNA, 1.04 AU/creatinine ± 0.58 AU/creatinine). The serum creatinine level, a marker of glomerulofiltration, was significantly lower in mice administered MLL3 shRNA than in those given control shRNA in the LPS model (control shRNA, 2.06 mg/dL ± 0.50 mg/dL; MLL3 shRNA, 1.40 mg/dL ± 0.40 mg/dL). Thus, MLL3 shRNA not only decreased LPS-induced increases in H3K4 me3 but also ameliorated the renal dysfunction and albuminuria.

### Examination using cultured podocytes

The effects of MLL3 shRNA were examined using cultured mouse podocytes. Mouse podocyte cells (E11 cell) cultured in a basal medium with 10% FCS were treated with MLL3 shRNA or control shRNA. The expression levels of H3K4 me3 were determined by a sandwich ELISA, and the expression levels of cathepsin L and synaptopodin were analyzed by Western blot.

**Fig. 9:**
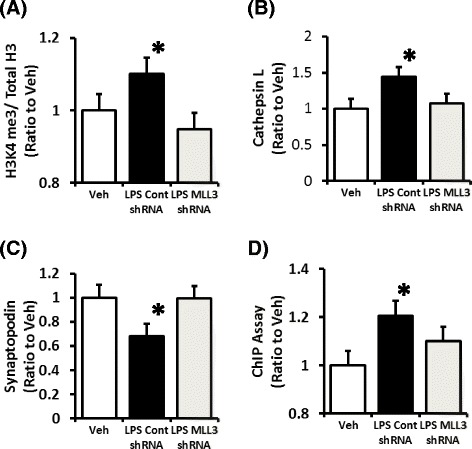
Effect of MLL3 shRNA on synaptopodin and cathepsin L expression and histone H3K4 me3 levels and the determination of H3K4 me3 level on the promoter region of cathepsin L in cultured mouse podocytes co-cultured with LPS-stimulated peritoneal macrophages. In podocytes co-cultured with peritoneal macrophages stimulated with LPS, increased H3K4 me3 was found compared to untreated podocytes (Veh) (**a**). MLL3 shRNA administration decreased H3K4 me3 compared to that of cells administered with control shRNA (**a**). MLL3 shRNA administration also decreased the level of cathepsin L protein expression compared to that of cells administered the control shRNA (**b**). MLL3 shRNA increased the expression of synaptopodin compared to that of the cells administered control shRNA in cultures stimulated with cytokines derived from co-cultured macrophages (**c**). In chromatin immunoprecipitation (ChIP) assays with E11 cells stimulated with cytokines derived from co-cultured macrophages, the stimulated levels of H3K4 me3 at the cathepsin L promoters were significantly lower in E11 cells treated with MLL3 shRNA 72 h post-administration compared with the control shRNA (**d**). *P < 0.05 vs. Veh. Veh, untreated podocytes; Cont, control shRNA; H3K4 me3, histone H3K4 trimethylation; total H3, total histone H3; *N* = 4. Data are expressed as the mean ± SE

The effects of LPS were examined to cause increased H3K4 methylation in podocytes. LPS did not directly cause increased H3K4 methylation in the cultured podocytes. In the co-cultured podocytes with peritoneal macrophages stimulated with LPS, increased H3K4 me3 was shown in podocytes compared with the unstimulated cells (Fig. 9a). These data suggest that cytokines derived from macrophages stimulated with LPS cause increased H3K4 me3 in podocytes. We treated MLL3 shRNA-transduced cultured podocytes and compared them to the control shRNA transduced podocytes followed by analysis of H3K4 methylation under these conditions. In the samples extracted from E11 cells stimulated with cytokines derived from macrophages using a co-cultured system that followed the acid extraction method and sandwich ELISA, MLL3 shRNA administration decreased H3K4 me3 compared to that of cells administered the control shRNA (Fig. 9a). MLL3 shRNA administration decreased the cathepsin L protein expression compared to that of cells administered with control shRNA (Fig. 9b). In addition, in the cultures stimulated with cytokines derived from co-cultured macrophages, MLL3 shRNA increased the expression of synaptopodin compared with to that of cells administered control shRNA (Fig. 9c). In chromatin immunoprecipitation (ChIP) assays in E11 cells stimulated with cytokines derived from co-cultured macrophages, levels of H3K4 me3 at the cathepsin L promoters were significantly lower in E11 cells treated with MLL3 shRNA after 72 h administration compared with the control shRNA (Fig. 9d).

## Discussion

Nephrotic proteinuria associated with MN reflects a profound podocyte dysfunction; however, the pathogenic mechanism underlying this disorder remains unclear. We used immunohistochemical staining and electron microscopy to investigate the renal biopsies from six patients with nephrotic syndrome and PLA2R1-positive primary MN. We observed the co-localization of H3K4 me3 and the podocyte marker WT-1. The overlapping expression of H3K4 me3 and cathepsin L in patients with MN was significantly elevated compared with the controls. Elevated H3K4 me3 in patients with MN was associated with the downregulation of synaptopodin and positively associated with proteinuria. However, patient characteristics, including the mean age, differed significantly between the groups. The similarity in sex and age of patients with MN in this study may contribute to the observed relationship between histone H3K4 me3 and proteinuria or the expression of synaptopodin.

We also observed that proteinuria and renal dysfunction in an LPS-induced murine model of the disease was accompanied by an increase in histone H3K4 me3 and cathepsin L protein, decreased levels of synaptopodin protein, and an increase in podocyte swelling. These features were restored by MLL3 shRNA administration. The high efficacy of an in vivo transfection may result from the increased filtration in the basement membrane of the LPS model, in addition to the use of a conjugate-containing anti-nephrin antibody that recognizes the extracellular domain of nephrin in podocytes. Moreover, it may allow nephrin internalization to an intracellular site following its dislocation from the plasma membrane.

The effects of MLL3 shRNA were examined using the mouse podocytes under the condition stimulated with cytokines derived from co-cultured macrophages. MLL3 shRNA administration decreased H3K4 me3 compared to that of E11 cells administered with the control shRNA as determined by acid extraction followed by a sandwich ELISA. MLL3 shRNA administration increased the expression of synaptopodin compared to that administered with the control shRNA. MLL3 shRNA administration decreased cathepsin L protein expression compared to that of cells administered with the control shRNA. Levels of H3K4 me3 at the cathepsin L promoters were significantly lower in E11 cells treated with MLL3 shRNA than in those treated with the control shRNA. Although these data do not exclude the possibility that H3K4 methylation may occur in mesangial cells and endothelial cell nuclei, these data support an important role for epigenetic H3K4 me3 modification in the podocytes of kidney glomeruli.

Our results indicate a relationship between H3K4 me3, proteinuria, and synaptopodin with respect to MN pathophysiology. We observed increased H3K4 me3, enhanced cathepsin L, and decreased synaptopodin expression in the kidney podocytes of mice treated with LPS compared to that of the controls. The significance of the induction of H3K4 me3 in the upregulation of cathepsin L is further underscored by the finding that H3K4 me3 induces cathepsin L transcription as measured by a ChIP assay and real-time RT-PCR in both the LPS model and cultured podocytes. Cathepsin L is a potent endoprotease primarily responsible for the final breakdown of proteins within lysosomal compartments [12]. The importance of podocyte cathepsin L is highlighted by both in vitro data and animal models of glomerular diseases, as well as expression studies of human biopsies [8, 13, 14]. Two substrates of cytosolic cathepsin L have been described in podocytes: 1) dynamin [14] and 2) synaptopodin [11, 15]. Both of these proteins contribute to the F-actin structure in normal podocyte foot processes by promoting foot process effacement following the enzymatic processing by cathepsin L. Synaptopodin-deficient mice display impaired recovery from protamine-sulfate-induced foot process effacement and LPS-induced nephrotic syndrome [16]. A recent study reported that the beneficial effect of cyclosporine A (CsA) on proteinuria results from the stabilization of the actin cytoskeleton in kidney podocytes [11]. CsA blocks the calcineurin-mediated dephosphorylation of synaptopodin, thereby preserving the phosphorylation-dependent synaptopodin/14-3-3b interaction [17]. Preservation of this interaction protects synaptopodin from cathepsin-L-mediated degradation. A recent study investigated the effects of targeting dynamin’s oligomerization cycle with Bis-T-23, which promotes actin-dependent dynamin oligomerization in whole animals [18]. Bis-T-23 ameliorated or prevented proteinuria and diminished the mesangial matrix expansion in genetic and chronic models of glomerular disease in rodents. This study suggests the feasibility of treating a diverse range of glomerular kidney diseases by modulating actin dynamics. In light of these findings, our observation that H3K4 me3 is associated with the downregulation of synaptopodin in podocytes. Moreover, the proteinuria in patients with MN can be explained by the increased degradation of synaptopodin due to the upregulation of cathepsin L. Proteinuria in an LPS-induced murine model and patients with MN may result from changes in podocyte morphology and motility following alterations in the foot-process architecture. Strategies to protect synaptopodin from degradation may provide a promising starting point for the development of antiproteinuric drugs.

We observed that the LPS-induced proteinuria, impaired renal function, and altered expression of H3K4 me3, cathepsin L, and synaptopodin were restored by the administration of shRNA against MLL3, a component of the histone H3K4 methyltransferase complex. This result confirms the involvement of H3K4 methylation in the mechanism of LPS-induced proteinuria, as the recruitment of MLL3 to the chromatinized cathepsin L gene is known to mediate histone methylation and its subsequent gene expression. This result was confirmed in cultured podocytes. Dysregulation of MLL3 activity is associated with multiple cancers [19–21], and the targeted inactivation of MLL3 H3K4 methylation in mice results in ureter epithelial tumors [22]. These findings suggest that specific agonists/antagonists of MLL3/4 H3K4MT activity could be useful for treating a variety of disorders. Similar to MLL3, PTIP is a component of the histone H3K4 methyltransferase complex. This protein is required for the enzymatic activity of the complex. PTIP knockout mice demonstrate podocyte dysfunction [23], suggesting a maintenance function for PTIP-mediated H3K4 me3 in podocytes. Urine albumin levels of PTIP knockout mice were 10- to 30-fold higher than those of WT mice. Moreover, the podocyte foot processes of the knockout mice appeared much more irregular and flattened under electron microscopy. This observation is in contrast to our finding that an MLL3 knockdown ameliorated the effects of LPS by suppressing H3K4 methylation. We speculate that these contrasting results stem from differences in the intensity of H3K4 me3 inhibition. A previous report showed that MLL3 interacts with transcription factors and facilitates the recruitment of the PTIP complex to target gene promoters [24]. We further observed that the downregulation of MLL3 results in changes in the transcriptional profile of terminally differentiated podocyte cells, which ultimately protects against the glomerular disease phenotype induced by LPS. Our results demonstrate a function for MLL3-mediated H3K4 methylation, and indicate a novel role for H3K4 me3 in podocyte motility.

The median age of onset of MN is in the early 50s, an MN has been established as the most important cause of the nephrotic syndrome in elderly patients (aged > 65 years). Accumulating evidence also suggests that aging is associated with systemic and vascular inflammation [25]. Circulating inflammatory cytokines are elevated in some older adults [26, 27], and chronic exposure to inflammation is thought to be closely related to the development and progression of renal injury [28–30]. Previous studies have shown that the LPS-mediated NF-kB signaling pathway, which plays a critical role in the inflammatory response, is involved in H3K4 me3 redistribution in monocyte-derived dendritic cells [31, 32]. Silencing of MLL inhibits both H3K4 me3 enrichment and miRNA expression, and affects TNF-α production [31, 32]. Our data using a co-cultured system with podocytes and peritoneal macrophages suggests that cytokines derived from macrophages stimulated with LPS cause increased H3K4 me3 in podocytes. Thus, MN pathogenesis may involve the induction of H3K4 me3 by inflammatory cytokines, leading to an increased expression of cathepsin L and a decreased expression of synaptopodin. These changes were observed to induce the deterioration of renal function and proteinuria in LPS-treated mice, and were consistent with the findings in patients with MN.

### Study limitations

There were several limitations in the current study. Firstly, age-matched control samples from healthy donors were not obtained in a series of consecutive patients and there is difference in the methods for preparation of fixation and embedding. Moreover, there was a difference in the serum creatinine levels and mean age between MN and minimal change, FSGS, or amyloidosis. Finally, the LPS-induced murine model is not a suitable model for MN. Other models, including the cationic BSA injection or heymann nephritis model will also need to be examined in the future.

## Conclusions

Epigenetic H3K4 me3 modification at the cathepsin L promoter increases cathepsin L expression and is associated with podocyte dysfunction and MN pathophysiology. Therefore, targeting this epigenetic signature of histone H3K4 me3 may be an effective strategy to ameliorate the consequences of MN.

## Abbreviations

H3K4 me3: Histone H3K4 trimethylation
MN: Membranous nephropathy
